# Energy-efficient framework based on optimal antenna selection in S-NOMA supported UAV IoT networks

**DOI:** 10.1371/journal.pone.0337759

**Published:** 2026-01-02

**Authors:** Lav Soni, Ashu Taneja, Nayef Alqahtani, Ali Alqahtani

**Affiliations:** 1 Chitkara University Institute of Engineering and Technology, Chitkara University, Punjab, India; 2 Department of Electrical Engineering, College of Engineering, King Faisal University, Al-Ahsa, Saudi Arabia; 3 Department of Networks and Communications Engineering, College of Computer Science and Information Systems, Najran University, Najran, Saudi Arabia; Guangdong University of Petrochemical Technology, CHINA

## Abstract

Owing to the high emissions and increased energy consumption of the expanding heterogeneous internet-of-things (IoT) devices across terrestial and non-terrestial networks, achieving the energy sustainability in future IoT networks is the main challenge. This paper presents an energy efficient framework utilising spatial non orthogonal multiple access (S-NOMA) technique in UAV assisted IoT networks. An antenna selection algorithm is proposed that selects a set of active antennas enabling user fairness. The numerical formulations for the air-to-ground communication links in the S-NOMA system is also obtained. Further, the paper proposes a power consumption model for the S-NOMA enabled network to carry out the energy efficiency analysis. The transmit power consumption, circuit power consumption and UAV hovering power is taken into account. The proposed S-NOMA framework with optimal antenna selection is evaluated against conventional NOMA and random schemes. Simulation results demonstrate that S-NOMA achieves superior performance in terms of data rate and energy efficiency. It is observed that at an SNR of 30 dB, the proposed method with achieves a data rate of 15.2 bps/Hz, outperforming conventional NOMA which achieves 6.4 bps/Hz. Also, the energy efficiency improves by 14.4% at transmit power *P*=25 dBm with the proposed antenna selection scheme over random selection scheme. This improvement is attributed to the enhanced spatial gain and power-aware antenna selection, thus resulting in sustainable UAV IoT networks.

## 1 Introduction

### 1.1 Need and motivation

To support the densification and heterogeneity of Internet-of-things (IoT) services, increasing number of smartphones, extended reality (XR) devices, ground or aerial users, efficient and reliable communication networks are envisioned [1]. Sixth generation (6G) technology aims to offer fully connected world with full coverage connectivity between users, vehicles, sensors, smart devices, data and cloud processors and network resources [2]. 6G supports diverse IoT applications including smart cities, agriculture, healthcare, and industrial automation, which require real-time data exchange between devices with varying communication and energy requirements [3]. However, the major challenge facing the 6G IoT network is managing the energy consumption and communication efficiency of devices, especially in environments where devices are resource-constrained [4]. The high emissions and increased energy consumption are the main barriers for sustainable IoT network [5].

6G shows great promise for supporting drones and satellites as well as terrestrial wireless infrastructures [6]. Unmanned aerial vehicles (UAVs) or drones can provide IoT device coverage, connection, and data relay services in places with insufficient infrastructure or where traditional communication techniques fail [7]. Miniature UAVs can serve as mobile base stations, creating a flexible communication infrastructure [8]. UAVs’ line-of-sight (LoS) connectivity are crucial in challenging situations. Enhanced system capacity and spectral efficiency have great potential in 6G networks. However, the effectiveness of UAV-assisted IoT networks depends on the communication technology, power needs, and UAV energy limits [9].

### 1.2 Massive radio access support

To enable large IoT access, UAVs are crucial. UAVs capture sensory data from diverse areas and send it to cloud servers for effective decision-making. This requires 6G enabling solutions for large radio access support [10]. Due to its multi-access capability, non-orthogonal multiple access (NOMA) is commonly employed in 6G architectural paradigms when orthogonal multiple access (OMA) cannot handle huge access [11].

NOMA lets numerous users to share frequency and temporal resources by superimposing their signals at varied power levels, unlike previous OMA methods [12]. This improves spectrum utilisation, especially in high user density or bandwidth-constrained networks. NOMA optimises power allocation and serves various devices in UAV-assisted IoT networks [13]. Efficient power allocation enhances signal-to-noise ratio (SNR), reduces interference, and manages device energy consumption. In UAV-assisted IoT networks, power allocation solutions are critical due to limited UAV energy resources [14]. A smart power allocation technique can extend UAV operation and meet IoT device communication needs. Proper power regulation allows stable device connections in tough circumstances, such as high elevations or urban canyons, where signal propagation may be impacted [15]. Thus, a unified architecture that maximizes energy economy and communication performance of UAV-assisted IoT networks is essential due to rising IoT device demand and the necessity for continuous connectivity [16]. Intelligent multiple access and power allocation solutions help solve next-generation IoT application energy consumption, coverage and network scalability issues [17]. OMA allocates orthogonal resources, while NOMA improves efficiency by sharing them in the power domain. Spatial NOMA (S-NOMA) further enhances performance by exploiting the spatial domain. Their comparison on different aspects is shown in Table 1.

**Table 1:** Comparison between S-NOMA, NOMA and OMA based on different aspects.

Aspect	OMA (Orthogonal Multiple Access)	NOMA (Non-Orthogonal Multiple Access)	S-NOMA (Spatial NOMA)
Multiplexing domain	Time/Frequency/Code	Power domain multiplexing	Spatial
User separation	Users are separated in orthogonal time, frequency, or code resources	Users are superimposed by power levels.	Users are separated by spatial beams or precoding patterns.
Spectrum efficiency	Moderate, limited by orthogonality	High, multiple users share the same resource block	Very high, scales with number of spatial streams
Receiver complexity	Low	Medium to high owing to SIC requirement	Medium, require accurate CSI and spatial processing
Scalability	Limited, one user per block	Multiple users per block	Many users across space & power
Energy Efficiency	Moderate	Reduced (SIC overhead)	High with optimized design
Latency	Low	Moderate, due to SIC processing	Low to moderate, depends on beamforming complexity
Deployment	4G/LTE, Wi-Fi	5G	6G, massive MIMO, UAVs

### 1.3 Related work

This subsection presents the recent research works carried out pertaining to the performance analysis of UAV networks in multi-tier communication domains. The literature includes number of papers, for example, the throughput performance of UAV-aided multiuser terahertz (THz) systems is analyzed in [18]. It employs NOMA and hybrid automatic repeat request (HARQ) protocols and the study accounts for path loss, fog attenuation and imperfect CSI. Simulation results demonstrate significant throughput improvements over existing methods. The authors in [19] investigates antenna selection in downlink MIMO-NOMA systems with multi-antenna base stations and users. An iterative antenna selection scheme with a power estimation method is proposed for two-user systems and extended to multiuser scenarios. Numerical results show the algorithm achieves near-optimal performance with significantly reduced computational complexity. A secure reconfigurable intelligent surfaces (RIS) assisted UAV multiuser massive MIMO-OFDM system is proposed in [20].

The spectral efficiency is enhanced using frequency domain spectral shaping (FDSS) and discrete cosine transform (DCT) spread which reduce out-of-band emissions and cut multiuser interference. Incorporating physical layer security with block diagonalization, channel coding, and advanced detection, simulations show notable gains in spectral efficiency and reduction in bit error rate (BER), confirming the framework’s effectiveness for secure UAV wireless communications. The authors in [26] studied a NOMA-based downlink integrated satellite-terrestrial relay network (ISTRN) under hardware impairments (HI) and time-varying links. The radio frequency (RF) and free space optical (FSO) links are modeled by shadowed-Rician and Malaga fading distributions, respectively. Closed-form expressions for ergodic capacity of far and near users are derived and validated via Monte Carlo simulations. The impact of HI, wind speed, zenith angle, and power allocation on capacity is also examined. An air-to-ground network is proposed in [27] where UAV base stations move in circular orbits to enhance energy efficiency and network stability. The orbit arrangement is optimized in two steps: minimizing coverage holes and maximizing capacity, then adjusting UAV speeds to maximize energy efficiency based on user density. Simulation results validate network’s improved performance. The authors [28] study a UAV-enabled massive MIMO-NOMA full-duplex two-way relay system with low-resolution ADCs/DACs serving multi-pair ground users. They derive closed-form expressions for sum spectral and energy efficiency considering imperfect channel state information (CSI), successive interference cancellation (SIC) and quantization noise. Asymptotic analysis and power scaling laws evaluate parameter effects, and an optimization scheme maximizes spectral efficiency. Results show large-scale antennas and power control effectively reduce interference, UAV height impacts performance, and quantization bits influence the SE/EE trade-off, which varies with the Rician factor. The authors in [29] propose a wireless caching network combining UAV, millimeter wave (mmWave) MIMO, and NOMA to minimize user delay. They decompose the problem into UAV deployment, hybrid beamforming, and power allocation. K-means and user pairing optimize UAV-user proximity and channel gain gap, while PSO and zero forcing handle analog and digital beamforming. Genetic algorithm optimizes NOMA power allocation. Simulations show the proposed scheme reduces user delay compared to baselines. This work [30] investigates a collaborative sensing system where multiple UAVs transmit data to a cloud server using a cell-free MIMO network to manage inter-UAV interference. A hybrid transmission strategy combining TDMA, NOMA, and cooperative transmission is proposed. The joint optimization of task splitting and transmission strategy aims to minimize mission completion time. Numerical results demonstrate the effectiveness of the proposed approach in accelerating sensing missions. Table 2 presents the comparison of existing approaches with the proposed approach. The technologies, system models, performance parameters and key findings of existing approaches are discussed along with the challenges. In comparison, the proposed S-NOMA based UAV IoT framework demonstrates improved data rate and energy efficiency through power-aware antenna selection.

**Table 2:** Comparative analysis of proposed work over existing research.

Ref.	Technology	Performance Parameters	System Model	Research Findings	Challenges
[21]	Distributed MIMO	Estimation accuracy, noise handling, calibration robustness	Uncalibrated distributed MIMO radar with unknown noise and correlation	Proposes three estimators: relaxed MLE, Kronecker-constrained projection, and alternating minimization using biconvexity	Handling uncalibrated systems with complex Kronecker-structured noise
[22]	Semantic Communication with NOMA	Semantic transmission quality, adaptability, interference resilience	Semantic adaptive feature extraction (SAFE) network	SAFE with sub-semantics extraction, three refinement algorithms, and adaptive NOMA-based transmission	Adapting semantic transmission to varying and interfering wireless channels
[23]	NOMA-aided UAV Communication	Outage Probability (OP), Intercept Probability (IP), Secrecy Performance	Dual-hop NOMA UAV with trusted near user and untrusted far user	XOR-encrypted dual-hop scheme with exact and asymptotic OP/IP analysis	Lack of security against passive eavesdropping
[24]	UAV, Mobile Edge Computing (MEC), IoT	IoTs served %, User satisfaction, SP profit, UAV utilization	UAV-assisted collaborative MEC architecture for urban IoT offloading	TMSC offloading + K-means UAV placement; +19% IoTs served, +12% satisfaction, +25% profit with 25% fewer UAVs	Urban complexity, high IoT density, limited coverage
[25]	MIMO DFRC in a Satellite–UAV framework	SCNR, MUI Energy, QoS, Spectral Efficiency, Clutter Suppression	Joint waveform–filter design via STAP under spectral, communication, and CM constraints	Proposes a nonconvex optimization solution using cyclic optimization, Dinkelbach’s transform, and ADMM	Widely distributed clutter, MUI suppression, multispectral coexistence, complexity
This work	S-NOMA in UAV-assisted IoT	Data rate, Energy efficiency, Mutual information	Air-to-ground S-NOMA with antenna selection & power consumption	15.2 bps/Hz @30 dB vs 6.4 (NOMA); 14.4% higher EE @25 dB; due to spatial gain & power-aware selection	UAV energy limits, perfect CSI assumption

### 1.4 Contributions and outcomes

As IoT continues to evolve into its next generation, the exponential growth in the number of devices, sensors, and communication modules has led to massive data exchange and increased energy demands. This results in network congestion, packet losses, and communication inefficiencies, especially in remote or underserved areas. Miniature UAVs have emerged as a promising solution to enhance coverage and support data transmission in such challenging environments. However, due to limited onboard energy and processing capacity, the design of an efficient communication and power allocation scheme becomes critical. This paper addresses these challenges by proposing an energy efficient framework for S-NOMA enabled UAV assisted IoT networks. The main contributions of this work are given below:

- To address energy constraints and interference management in dense IoT deployments, the paper introduces a Spatial NOMA (S-NOMA) scheme that utilizes multi-antenna UAVs combined with transmit antenna selection (TAS). The S-NOMA technique leverages power-domain multiplexing and spatial diversity to serve multiple users concurrently, thus maximizing spectral efficiency.
- The mathematical formulations for the channel model incorporating both line-of-sight (LoS) and non-line-of-sight (NLoS) components for the air-to-ground (A2G) communication links are obtained providing a realistic modeling framework.
- An antenna selection algorithm is proposed that selects the set of active antennas ensuring fairness among users and maximizing mutual information.
- The energy efficiency (EE) analysis is carried out utilising the proposed power consumption model that includes transmit power *P*_*t*_, circuit power *P*_*cir*_, and UAV hovering power *P*_*h*_. EE, measured in bits-per-joule, is optimized by adjusting the power allocation coefficients.
- The system evaluation under varying UAV altitudes, transmit antennas (*N*_*T*_), and power allocation coefficients ($\alpha_{j}$) is also presented.
- In the end, the comparison between S-NOMA and conventional NOMA systems is discussed for proposed antenna selection scheme and traditional random antenna selection scheme to validate the effectiveness of the former over the latter.

## 2 System model

**Fig 1:**
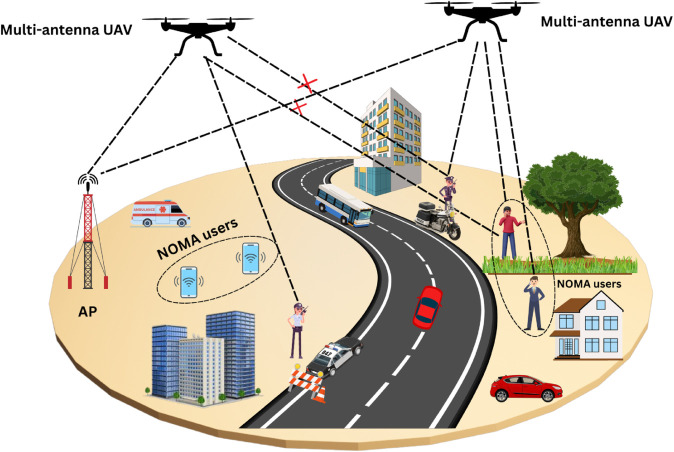
A communication model of UAV assisted IoT network serving diverse set of users.

Consider a UAV aided IoT network in which a UAV acting as aerial base station (BS) communicates with number of users or IoT nodes. The UAV is equipped with *N*_*T*_ antennas while the IoT users are having single receive antenna *N*_*R*_. The UAV provides line-of-sight (LoS) links to the user communication to enhance the coverage. The users are grouped following non-orthogonal multiple access (NOMA) principle with power domain multiplexing to manage interference. The downlink scenario is considered in which out of *N*_*T*_ UAV antennas, only one antenna is active in a given time slot, *N*_*A*_ = 1. One set of NOMA users in one spatial direction are served by one beam while another set is served by another beam following spatial NOMA (S-NOMA) using multi-antenna UAV. The proposed S-NOMA scheme primarily considers single active-antenna transmission consistent with spatial modulation. Fig 1 shows a UAV assisted IoT network in which a diverse set of users ranging from mobile devices to IoT nodes operate within the UAV’s coverage area. The UAV’s transmitted signal consists of two main components: the first is transmitted directly through the spatial domain via the selected active antenna, while the second is formed by superimposing multiple user signals in accordance with NOMA principles.

### 2.1 Proposed S-NOMA

The operational framework of the proposed S-NOMA scheme involves users within a group labeled as *U*_1_ to *U*_*k*_. A subset of each user’s bits is allocated for determining the active transmit antenna. Given *N*_*T*_ transmit antennas at the UAV, the number of bits used for transmit antenna selection (TAS) is less than $log_{2}(N_{T})$. The remaining bits are combined using power-domain NOMA to form a superimposed signal, which is then transmitted via the selected antenna. At the receiver side, signal detection is performed using a combination of maximum likelihood (ML) detection and successive interference cancellation (SIC). Through the integration of spatial diversity and NOMA principles, the proposed S-NOMA technique provides enhanced spectral efficiency and performance gains for all users in the network.

### 2.2 Channel modelling

The modelling of air-to-ground (A2G) downlink UAV channel is presented here. These variations are primarily influenced by the UAV’s altitude and the angle of elevation relative to the users. Both line-of-sight (LoS) and non-line-of-sight (NLoS) components are considered in modeling the A2G link. The overall channel matrix can be represented as:

$$𝐡=\sqrt{\frac{\kappa }{\kappa +1}}{\hat{𝐡}}_{LoS}+\sqrt{\frac{1}{\kappa +1}}{\tilde{𝐡}}_{NLoS},$$ (1)

where ${\hat{𝐡}}_{LoS}$ denotes the deterministic LoS component, and ${\tilde{𝐡}}_{NLoS}$ captures the random NLoS variations [31]. κ is the rician factor. Following the model, the NLoS component can be expressed as:

$${\tilde{𝐡}}_{NLoS}={𝐑}^{1/2}\bar{𝐡}{𝐓}^{1/2},$$ (2)

where $\bar{𝐡}$ is an independent Rayleigh fading matrix. $𝐑\in {\mathbb{C}}^{N_{R}\times N_{R}}$ and $𝐓\in {\mathbb{C}}^{N_{T}\times N_{T}}$ represent the receive and transmit correlation matrices, respectively. Their entries are defined as $[𝐑{]}_{p,q}=\lambda_{r}^{\left|p-q\right|}$ and $[𝐓{]}_{\hat{p},\hat{q}}=\lambda_{t}^{\left|\hat{p}-\hat{q}\right|}$, where $\lambda_{r}$ and $\lambda_{t}$ are spatial correlation coefficients. The distance between the UAV and the *j*^*th*^ ground user can be calculated by projecting the UAV onto the horizontal plane:

$$d_{j}=\sqrt{h_{UAV}^{2}+r_{j}^{2}},$$ (3)

where $h_{UAV}$ is the UAV altitude and *r*_*j*_ is the horizontal distance between the user and the UAV’s projection point on the ground [32]. The corresponding elevation angle is given by:

$$\psi_{j}=\arctan\left(\frac{h_{UAV}}{r_{j}}\right).$$ (4)

The probability of establishing a LoS link is modeled as:

$$p_{\text{LoS}}(\psi_{j})=\varsigma_{1}(\psi_{j}-\psi_{0}{)}^{\varsigma_{2}},$$ (5)

The NLoS probability is given by:

$$p_{\text{NLoS}}(\psi_{j})=1-p_{\text{LoS}}(\psi_{j}).$$ (6)

where $\varsigma_{1}$ and $\varsigma_{2}$ are empirical constants that depend on the environment and the operating frequency. $\psi_{0}$ is a reference elevation angle, typically set to $15^{\circ }$ [33].

The path loss values for LoS and NLoS links are modeled as:

$$\rho_{1}=a_{1}e^{-b_{1}\psi_{j}},\;\rho_{2}=a_{2}e^{-b_{2}\psi_{j}},$$ (7)

where *a* and *b* are frequency and environment-dependent constants. Taking UAV mobility into account, the instantaneous large-scale path loss (in dB) is modeled as the weighted average of LoS and NLoS components:

$$\rho_{0}=p_{\text{LoS}}(\psi_{j})\;\cdot \;\rho_{1}+p_{\text{NLoS}}(\psi_{j})\;\cdot \;\rho_{2}$$ (8)

This expression clearly shows that the path loss $\rho_{0}$ is influenced by several factors, including UAV altitude, user distance, carrier frequency, and environmental conditions. As the UAV moves or adjusts its altitude, the path loss dynamically varies with time.

### 2.3 Signal modelling

The signal modelling of the proposed scheme is presented here. Each user can act as active data user whose bits are utilised for antenna selection (AS). The first $\log_{2}(N_{T})$ bits of a user are used for transmit antenna selection (TAS). Let us suppose *n*_*b*_ be the total number of AS bits given by

$$n_{b}=[n_{b1},n_{b2},\ldots ,n_{bk}]$$ (9)

where *n*_*bj*_ are the TAS bits of the *j*^*th*^ user such that j∈{1,2,…,k}, and *k* is the number of users in the coverage area. The signal transmitted from the selected antenna is a power-domain superimposed signal of all users:

$$x=\sum_{j=1}^{k}\sqrt{\alpha_{j}}s_{j}$$ (10)

where $\alpha_{j}$ is the power allocation coefficient for the *j*^*th*^ user, and $\sum_{j=1}^{m}\alpha_{j}\leq 1$. We assume $𝔼[s_{j}^{2}]=E_{s}$ for each user.

The received signal at the *j*^*th*^ user is:

$$y_{j}=h_{j}I_{n_{b}}\sum_{j=1}^{k}\sqrt{\alpha_{j}}s_{j}+z_{j}$$ (11)

Here, ${𝐡}_{j}\in {\mathbb{C}}^{N_{R}\times N_{T}}$ is the channel matrix from UAV to user *j*, *s*_*j*_ is the signal of user *j*, $\alpha_{j}$ is the power allocation factor, $I_{n_{b}}$ is a column of the identity matrix indicating the selected transmit antenna, and ${𝐳}_{j}$ is complex additive white gaussian noise (AWGN) with power spectral density $\sigma_{0}^{2}$. The channel matrix is represented by ${𝐡}_{i,j}\in {\mathbb{C}}^{N_{R}\times 1}$, *i* denoting the selected antenna. In NOMA, less power is allocated to users with better channel state information (CSI) to maintain fairness such that

$$\left\|h_{i,1}\|<\|h_{i,2}\|<\ldots <\|h_{i,k}\right\|$$ (12)

the *k*^*th*^ user receives the least power. The user with the strong channel condition is allocated the least power, $\alpha_{k}P$ with *P* being the total transmitted power.

Using successive interference cancellation (SIC), the highest power signal is decoded first. For the first user, the detection is given as:

$$(n_{b1},s_{1})=\arg\min_{i,{\hat{s}}_{1}}\left[{\left\|y_{1}-h_{i,1}\alpha_{1}{\hat{s}}_{1}\right\|}^{2}\right]$$ (13)

This can be generalized for the *j*^*th*^ user as

$$(n_{bj},s_{j})=\arg\min_{i_{j},{\hat{s}}_{j}}{\left\|y_{j}-\left(\sum_{q=1}^{j-1}h_{i_{q},q}\alpha_{k}{\tilde{s}}_{k}\right)-h_{i_{j},j}\alpha_{j}{\hat{s}}_{j}\right\|}^{2}$$ (14)

where ${\hat{s}}_{j}$ is the estimated signal of user *j*, with perfect SIC ${\tilde{s}}_{k}$ denotes deterministic signal of user *k* and *i*_*j*_ represents possible antenna selections.

### 2.4 Sum rate analysis

The signal capacity of user *j* under NOMA is:

$$C_{y_{j}}=\log_{2}\left(1+\frac{P\alpha_{j}E_{s}𝔼[\|h_{i,j}{\|}^{2}]}{P\sum_{q=j+1}^{k}\alpha_{q}E_{s}𝔼[\|h_{i,j}{\|}^{2}]+\sigma_{0}^{2}}\right)$$ (15)

Thus, the total capacity of user *j* is

$$R_{y_{j}}=C_{y_{j}}+I(n_{bj};y_{j})$$ (16)

The total system sum-rate is

$$R=\sum_{j=1}^{k}R_{y_{j}}$$ (17)

where $I(n_{tj};y_{j})$ is the mutual information (MI) between the TAS bits and the received signal.

$$I(n_{bj};y_{j})=\sum_{i=1}^{{𝒜}_{j}}r_{j}\int p_{r}(y_{j}|n_{bj})\log_{2}\left(\frac{\tilde{p}(n_{bj}|y_{j})}{p_{r}(y_{j})}\right)dy_{j}$$ (18)

where ${𝒜}_{j}$ is the number of bits defined by user *j*, and $r_{j}=1/{𝒜}_{j}$, with:

$${𝒜}_{j}=\left\lfloor \frac{N_{T}}{2\left(\frac{k-1}{k}\right)\log_{2}N_{T}}\right\rfloor$$ (19)

The upper bound of MI is $\log_{2}({𝒜}_{j})$, simplified as $\log_{2}(N_{T})/k$. The posterior probability $\tilde{p}(n_{bj}|y_{j})$ is:

$$\tilde{p}(n_{bj}|y_{j})=\frac{p_{r}(y_{j}|n_{bj})}{\sum_{i=1}^{{𝒜}_{j}}r_{i}p_{r}(y_{j}|n_{bj})}$$ (20)

The likelihood $p_{r}(y_{j}|n_{tj})$ is:

$$p_{r}(y_{j}|n_{bj})=\frac{1}{\pi^{N_{R}}\det(\Gamma )}\exp\left(-y_{j}^{†}\Gamma^{-1}y_{j}\right)$$ (21)

with:

$$\Gamma =\sigma_{0}^{2}𝐈+Ph_{i,j}h_{i,j}^{†}$$ (22)

### 2.5 Imperfect SIC analysis

To model the imperfect SIC, a residual interference factor ξ∈[0,1] is introduced. When a signal is cancelled by SIC a fraction ξ of its power remains as interference. For user *j* with power allocation fraction $\alpha_{j}$, transmit power *P*, channel gain $||h_{ij}|{|}^{2}$ and noise power $\sigma_{0}^{2}$, the received signal at user *j* is

$$y_{j}=\underset{\text{desired}}{\underbrace{\sqrt{\alpha_{j}P}\;h_{j}\;s_{j}}}\;+\;\underset{\text{uncancelled inter.}}{\underbrace{\sum_{k>j}\sqrt{\alpha_{k}P}\;h_{k}\;s_{k}}}\;+\;\underset{\text{residual (imperfect SIC)}}{\underbrace{\xi \sum_{k<j}\sqrt{\alpha_{k}P}\;h_{k}\;s_{k}}}\;+\;z.$$ (23)

The signal capacity of user *j* is:

$$C_{y_{j}}=\log_{2}\left(1+\frac{P\alpha_{j}E_{s}𝔼[\|h_{i,j}{\|}^{2}]}{P\xi \sum_{q<j}\alpha_{q}E_{s}𝔼[\|h_{i,j}{\|}^{2}]+P\sum_{q=j+1}^{k}\alpha_{q}E_{s}𝔼[\|h_{i,j}{\|}^{2}]+\sigma_{0}^{2}}\right)$$ (24)

For ξ=0, the above formulations reduce to the ideal case of perfect SIC.

### 2.6 Power consumption and energy efficiency analysis

This section presents the energy efficiency analysis of the system scenario defined in Sect 2. The power consumed in the system is represented as:

$$P_{T}=P_{t}+P_{h}+P_{cir}$$ (25)

where *P*_*h*_ is the UAV hovering power. Also,

$$P_{t}=\sum_{j=1}^{k}\alpha_{j}P$$ (26)

and *P*_*cir*_ is the circuit power consumption owing to the associated circuitry. The energy efficiency (EE) is defined as in bits-per-joule is:

$$EE=\frac{\sum_{j=1}^{k}R_{y_{j}}}{P_{T}}$$ (27)

*EE Maximization*

Denoting the per user power allocation coefficients by $\alpha =\left\{\alpha_{1},\alpha_{2}....\alpha_{k}\right\}$, let us define a scalar $\theta =\sum_{j=1}^{k}\alpha_{j}$ that represents the total fraction of power used for data transmission. The energy efficiency maximization can be formulated as


$$\max_{\theta ,\alpha_{j}}\;EE(\alpha ,\theta )=\frac{\sum_{j=1}^{k}R_{y_{j}}(\alpha ,\theta )}{P_{t}(\alpha ,\theta )+P_{cir}+P_{h}}$$

subject to the constraints  

$$\left\{ \begin{array}{c} \theta =\sum_{j=1}^{k}\alpha_{j}\\ 0<\theta \leq 1\\ R_{j}\geq R_{j}^{\min} \end{array}\right.$$ (28)

where $P_{t}=\sum_{j=1}^{k}\alpha_{j}P$ and $R_{y_{j}}=C_{y_{j}}+I(n_{bj};y_{j})$

Since this is fractional non-convex problem, the same can be solved by applying Dinkelbach’s transform to convert the fractional objective to a sequence of parameterized subtractive problems iterate on λ until convergence.

$$\max_{\alpha ,\;\theta }\;\sum_{j}R_{y_{j}}-\lambda (P_{t}+P_{h}+P_{cir})$$ (29)

By applying sequential convex approximation (SCA) the non-convexity in the inner loop can be taken care to obtain the optimal value.

## 3 UAV antenna selection

In the UAV assisted IoT network, the IoT nodes are distributed widely with many nodes located at the dead zones or hard-to-reach areas. The multiple antennas at the UAV provide LoS links to serve the user communication and enhance the coverage. Antenna selection allows steering or focussing the energy towards specific user clusters. Since UAVs are power constrained, selecting few active antennas against all antennas helps in power savings and increase the UAV flight duration. This section presents the antenna selection in UAV-aided IoT communication network. The algorithm is presented for UAV antenna selection explaining the steps involved.

### 3.1 Proposed algorithm

**Fig 2:**
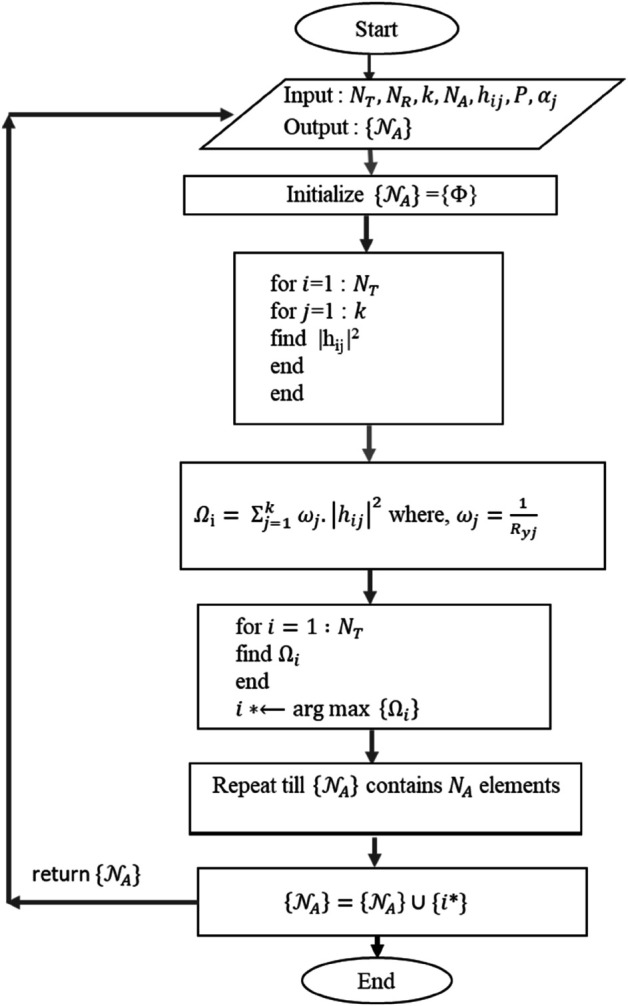
Flowchart of edge node selection algorithm.

The system model defined in Sect 2 contains *N*_*T*_ antenna UAV. The *k* users are grouped together defined by *U*_1_ to *U*_*k*_. Each user has *N*_*R*_ receive antennas. Out of *N*_*T*_ antennas on the UAV, *N*_*A*_ antennas are selected. Let us suppose $\left\{{𝒩}_{A}\right\}$ be the subset of active antennas at a given time slot. Initially the subset $\left\{{𝒩}_{A}\right\}$ is assigned a null set {Φ}. The first step is the measurement of channel gains between each UAV antenna *i* and each user *j* defined by $|h_{ij}{|}^{2}$. The metric for antenna selection, chosen as $\Omega_{i}=\sum_{j=1}^{k}w_{j}\;\cdot \;|h_{ij}{|}^{2}$ is obtained for each user *j*, where *w*_*j*_ is the user weight associated for selection. The antenna with the maximum value of selection metric $\Omega_{i}$ is selected, $i^{*}\leftarrow \arg\max\left\{\Omega_{i}\right\}$. The selected antenna i^*^ is added to the subset $\left\{{𝒩}_{A}\right\}$. The process is repeated till $\left\{{𝒩}_{A}\right\}$ contains *N*_*A*_ elements. Fig 2 shows the flowchart of the proposed algorithm.

**Algorithm 1 UAV antenna selection algorithm.**

**Input:** *N*_*T*_, *N*_*R*_, *k*, *N*_*A*_, *h*_*ij*_, *P*, $\alpha_{j}$

**Output:** $\left\{{𝒩}_{A}\right\}$

1. Initialize $\left\{{𝒩}_{A}\right\}=\left\{\Phi \right\}$

2. Measurement of channel gain

for *i* = 1 : *N*_*T*_

for j=1:k

find $|h_{ij}{|}^{2}$

end

end

3. Define selection metric

$\Omega_{i}=\sum_{j=1}^{k}w_{j}\;\cdot \;|h_{ij}{|}^{2}$

where, $w_{j}=\frac{1}{R_{y_{j}}}$

4. Selection of active antennas

for *i* = 1 : *N*_*T*_

find $\Omega_{i}$

end

$i^{*}\leftarrow \arg\max\left\{\Omega_{i}\right\}$

5. The subset $\left\{{𝒩}_{A}\right\}$ is updated with selected antenna i^*^

$\left\{{𝒩}_{A}\right\}=\left\{{𝒩}_{A}\right\}\cup \left\{i^{*}\right\}$

6. Repeat till $\left\{{𝒩}_{A}\right\}$ contains *N*_*A*_ elements

**return** $\left\{{𝒩}_{A}\right\}$

### 3.2 Computational complexity analysis

The proposed UAV antenna selection algorithm is evaluated for computational complexity in this subsection. A UAV assisted IoT communication network is considered which has *N*_*T*_ UAV antennas. The *k* users are grouped together which are denoted by *U*_1_ to *U*_*k*_. Out of *N*_*T*_ antennas, *N*_*A*_ antennas are selected. Initially, the algorithm computes the channel gains $|h_{ij}{|}^{2}$ between each UAV antenna *i* and each user *j*. Then, the metric for antenna selection, $\Omega_{i}=\sum_{j=1}^{k}w_{j}.{\left|h_{ij}\right|}^{2}$ is obtained for each user *j* which requires $O(N_{T}\;\cdot \;k)$ operations. The antenna with the maximum value of selection metric $\Omega_{i}$ is selected, $i^{*}\leftarrow \arg\max\left\{\Omega_{i}\right\}$ which requires *O*(*N*_*T*_) operations. The process repeats till *N*_*A*_ is chosen. Thus, the worst-case complexity is $O(N_{A}\;\cdot \;(N_{T}\;\cdot \;k\;+\;N_{T}))$. For small *N*_*T*_, second term becomes negligible, and the complexity becomes $O(N_{A}\;\cdot \;N_{T}\;\cdot \;k)$.

## 4 Results and discussions

This section presents the simulation results to validate the effectiveness of the proposed approach. The communication system scenario defined in Sect 2 is modelled in MATLAB. The simulation parameters used are listed in Table 3 [33,34]. The simulation is run over 10^4^ realizations which are averaged to obtain the data points. The study considers both urban and dense urban environments, with the air-to-ground path loss for the UAV channel calculated based on relevant channel characteristics. Without loss of generality, it is assumed that the antenna selection index is fairly allocated by the users. The energy efficiency (EE) performance of the proposed S-NOMA scheme is then compared with that of the conventional NOMA scheme.

**Table 3:** Description of the system parameters.

Parameter	Value	Parameter	Value
Frequency, *f*	2600 MHz	Minimum rate, *R*_*min*_	2 bits/s/Hz
UAV height, $h_{UAV}$	100 m	Reference elevation angle, $\psi_{0}$	$15^{\circ }$
Empirical constant, $\varsigma_{1}$	0.6	Empirical constant, $\varsigma_{2}$	0.11
Frequency constant, *a*_1_	10.39	Frequency constant, *a*_2_	29.6
Environment-dependent constant, *b*_1_	0.05	Environment-dependent constant, *b*_2_	0.03
Spatial correlation coefficient, $\lambda_{t}$	0.5	Spatial correlation coefficient, $\lambda_{r}$	0.5
Rician factor, κ	0.2	Noise variance, $\sigma_{0}^{2}$	10^−6^
Transmit antennas, *N*_*T*_	16	Receive antennas, *N*_*R*_	1
UAV hovering power, *P*_*h*_	20 dB	Circuit power, *P*_*cir*_	10 dB

**Fig 3:**
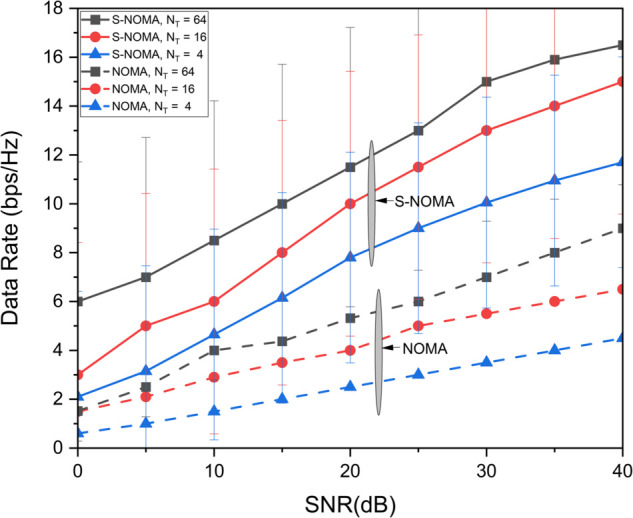
Data rate performance of NOMA and S-NOMA systems over different antenna configurations.

**Fig 4:**
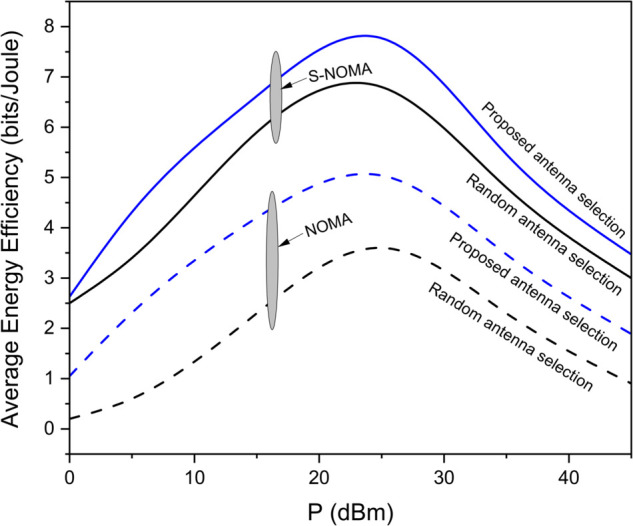
Average EE comparison of S-NOMA and conventional NOMA for different antenna selection schemes.

**Fig 5:**
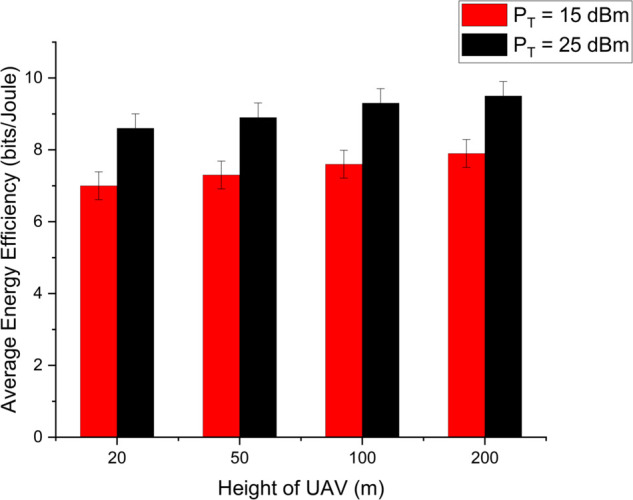
Comparison of system EE with different height of UAV over different transmit powers.

**Fig 6:**
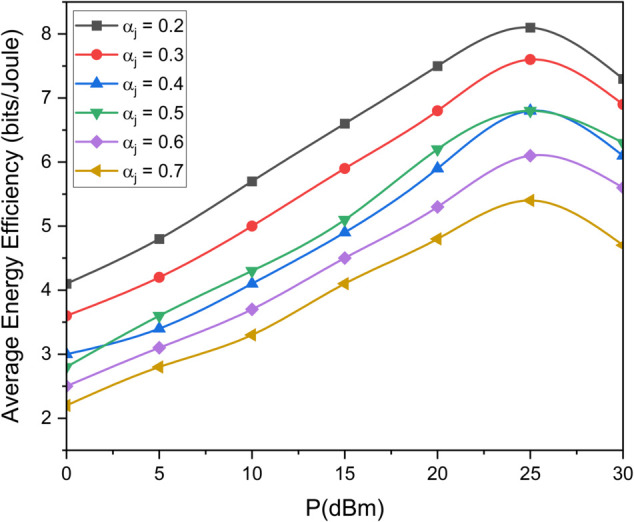
Comparison of EE for different power allocation factors.

**Fig 7:**
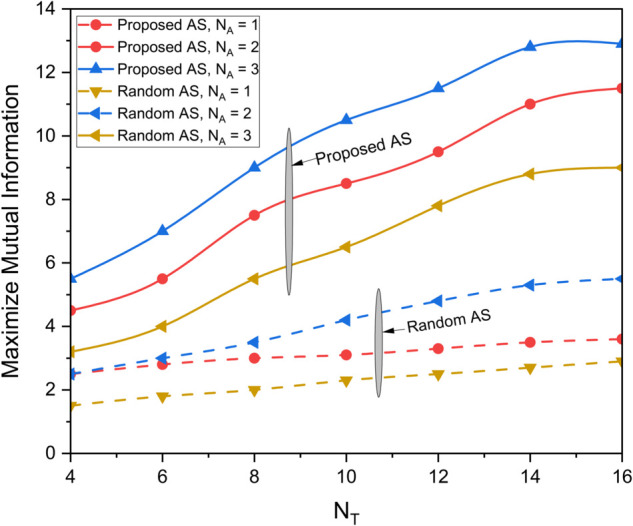
Maximum mutual information over varying *N*_*T*_ with random and proposed antenna selection.

Fig 3 illustrates the data rate performance comparison between S-NOMA and conventional NOMA schemes for different transmit antenna configurations (*N*_*T*_ = 4, 16, 64) over a range of SNR values from 0 to 40 dB. The results clearly demonstrate that S-NOMA consistently outperforms NOMA across all antenna configurations. For example, at SNR=30dB, S-NOMA with *N*_*T*_ = 64 achieves a data rate close to 15.2 bps/Hz, whereas NOMA with the same configuration attains 6.4 bps/Hz. This enhancement in performance is attributed to the superior spatial gain enabled by the S-NOMA scheme, particularly under the fair transmit antenna selection (TAS) strategy. The system benefits from maximum entropy, allowing for better utilization of spatial degrees of freedom. More *N*_*T*_ yields more data rate thereby increasing the performance. This equal allocation introduces maximum uncertainty, thereby increasing the achievable information rate. In contrast, NOMA owing to lack of spatiality has lesser performance. Fig 4 presents the average energy efficiency (EE) performance as a function of transmit power *P* (in dBm) for both the proposed and random antenna selection strategies under S-NOMA and NOMA schemes. As shown, the proposed antenna selection significantly outperforms random antenna selection across the entire transmit power range. For instance, at P=25dBm, the proposed method achieves an EE of 8 bits/Joule under S-NOMA, while the random selection under NOMA yields 3 bits/Joule. The improvement in energy efficiency is due to the intelligent selection of transmit antennas that optimizes spatial gain while minimizing unnecessary power consumption. The S-NOMA framework further enhances EE by allowing simultaneous transmission to multiple users on the same resource block, thereby improving spectral and energy utilization. The synergy of S-NOMA and optimized antenna selection proves to be highly effective for energy-constrained scenarios such as UAV-assisted IoT networks. Fig 5 illustrates the impact of UAV height on average energy efficiency at two different transmit power levels, *P*_*T*_ = 15 dBm and *P*_*T*_ = 25 dBm. As observed, EE initially increases with height and reaches a peak before starting to decline. Specifically, at a UAV height of approximately 100 meters, the EE is maximized, 9 bits/Joule for *P*_*T*_ = 15 dBm and 7 bits/Joule for *P*_*T*_ = 25 dBm. This behavior can be attributed to the trade-off between improved line-of-sight (LoS) probability at higher altitudes and increased path loss due to greater distance. At lower heights, poor channel conditions limit performance, while excessively high altitudes increase path loss, thereby reducing EE. An optimal UAV deployment height thus exists for maximizing energy efficiency, particularly in UAV-assisted communication networks. Fig 6 shows the effect of varying the power allocation coefficient $\alpha_{j}$ on the average energy efficiency. The analysis includes values of $\alpha_{j}=0.2,0.3,0.4,0.5,0.6,0.7$. The results reveal that EE increases with transmit power up to a certain point, after which it saturates or increases more slowly. When $\alpha_{j}$ is 0.2 less power is allocated to weak user thereby offering more power to strong user $(1\;-\;\alpha_{j})P$ resulting in increased EE. However, values too skewed toward either user (i.e., very low $\alpha_{j}$ or very high $\alpha_{j}$) result in suboptimal EE due to increased power imbalance and degraded decoding efficiency in the NOMA framework. This suggests that a balanced power allocation $\alpha_{j}=0.5$ between users provides the optimal trade-off between signal strength and interference management, leading to improved energy efficiency. Fig 7 presents the maximum mutual information (MI) as a function of the number of transmit antennas (*N*_*T*_), comparing the performance of the proposed and random antenna selection (AS) strategies for various numbers of active antennas (*N*_*A*_ = 1, 2, 3). The generalized case (*N*_*A*_ > 1), corresponds to generalized spatial modulation where multiple antennas can be active simultaneously. It shows the MI gain if multiple antennas are active using proposed antenna selection (AS) technique. The results demonstrate that the proposed AS consistently outperforms the random AS across all configurations and antenna counts.

Specifically, as *N*_*T*_ increases, the mutual information achieved with the proposed AS strategy grows significantly faster than that with random AS. For instance, with *N*_*A*_ = 3 and *N*_*T*_ = 16, the proposed AS achieves a mutual information close to 13 showing a gain of 18.18% over *N*_*A*_ = 2. Whereas the random AS with *N*_*A*_ = 3 and *N*_*T*_ = 16, has MI value less than 6. This substantial gain highlights the effectiveness of the proposed method in selecting the most informative antennas, thereby maximizing spatial-domain information throughput. The performance advantage becomes more pronounced with a higher number of active antennas, reinforcing the importance of intelligent antenna selection in enhancing the system’s spectral efficiency.

## 5 Conclusion

The main challenge in the UAV IoT networks is the fast energy drainage owing to the energy constained IoT devices resulting in increased power consumption. This paper addresses this challenge by proposing an energy-efficient S-NOMA enabled framework for UAV IoT networks. By leveraging spatial diversity and user fairness, an antenna selection algorithm is proposed that guarantees significant enhancement in both spectral and energy efficiency. The results reveal that with *N*_*T*_ = 64 and SNR of 25 dB, the proposed scheme achieves a data rate of up to 15.2 bps/Hz and energy efficiency of 8 bits/Joule, outperforming traditional strategies. The impact of UAV altitude on the energy efficiency performance suggests an optimal UAV altitude of around 100 meters balancing LoS probability and path loss. Furthermore, the analysis shows that a balanced power allocation coefficient $\alpha_{j}$ = 0.5 yields the best trade-off between interference management and signal strength. Mutual information also increases significantly with the number of active antennas *N*_*A*_ under the proposed selection scheme, confirming better spectral utilization compared to random selection. Overall, the integration of S-NOMA with proposed antenna selection presents a scalable and energy-aware communication framework, suitable for high-density, low-power UAV-assisted IoT deployments in future 6G wireless systems.
